# Sequence-assignment validation in cryo-EM models with *checkMySequence*

**DOI:** 10.1107/S2059798322005009

**Published:** 2022-06-07

**Authors:** Grzegorz Chojnowski

**Affiliations:** a European Molecular Biology Laboratory, Hamburg Unit, Notkestrasse 85, 22607 Hamburg, Germany

**Keywords:** cryo-EM, register shifts, sequence assignment, model validation, *checkMySequence*

## Abstract

The availability of new artificial intelligence-based protein-structure-prediction tools has radically changed the way that cryo-EM maps are interpreted, but it has not eliminated the challenges of map interpretation faced by a microscopist. Models will continue to be locally rebuilt and refined using interactive tools. This inevitably results in occasional errors, among which register shifts remain one of the most difficult to identify and correct. Here, *checkMySequence*, a fast, fully automated and parameter-free method for detecting register shifts in protein models built into cryo-EM maps, is introduced. It is shown that the method can assist model building in cases where poorer map resolution hinders visual interpretation. It is also shown that *checkMySequence* could have helped to avoid a widely discussed sequence-register error in a model of SARS-CoV-2 RNA-dependent RNA polymerase that was originally detected thanks to a visual residue-by-residue inspection by members of the structural biology community. The software is freely available at https://gitlab.com/gchojnowski/checkmysequence.

## Abstract 2

A new method, *checkMySequence*, for the fast and automated detection of register errors in protein models built into cryo-EM reconstructions is presented.

## 1. Introduction

Five years after the resolution revolution in cryogenic electron microscopy (cryo-EM) began, we are witnessing another revolution: in the accuracy of protein structure-prediction techniques. The former paved the way to the structure determination of large macromolecular complexes in the absence of crystals and at a level of detail that enabled the study of biological processes at the atomic scale (Kühlbrandt, 2014 ▸; Kokic *et al.*, 2021 ▸). The latter provided a means for the accurate and widely accessible structure prediction of protein structures. Although the release of the artificial intelligence-based *AlphaFold*2 (AF2; Jumper *et al.*, 2021 ▸) and the similar *RoseTTAFold* (Baek *et al.*, 2021 ▸) represent dramatic progress in the way that protein structures are predicted, they did not solve the problem of protein structure determination. Structural studies of multimeric, highly dynamic complexes or of specific ligand-bound states of proteins still require experimental approaches, and from this perspective the new structure-prediction approaches perfectly complement cryo-EM (Perrakis & Sixma, 2021 ▸). Nevertheless, the new predictive methods have dramatically changed the way that cryo-EM reconstructions are interpreted (Mosalaganti *et al.*, 2021 ▸).

With the availability of accurate protein structure-prediction tools, the interpretation of most cryo-EM reconstructions does not require *de novo* model tracing [for example, with *ARP*/*wARP* (Chojnowski *et al.*, 2021 ▸), *Buccaneer* (Hoh *et al.*, 2020 ▸) or *phenix.map_to_model* (Terwilliger *et al.*, 2018 ▸)] or parallel structure determination of the model components using X-ray crystallography (Beckham *et al.*, 2021 ▸). Instead, one can follow an approach that is often used in cryo-EM at lower resolutions, in which whole models are assembled from experimentally or theoretically determined structures of their components (Allegretti *et al.*, 2020 ▸). The structure-assembly procedure, however, does not eliminate the need for interactive (or ‘manual’) model rebuilding. This is still required in regions where a reliable structure cannot be predicted due to low sequence coverage or is predicted in a conformational state incompatible with a target (Perrakis & Sixma, 2021 ▸). Similarly, interfaces within homo- or hetero-multimers often cannot be reliably modelled even with the excellent, community-driven AF2 extension *ColabFold* (Mirdita *et al.*, 2021 ▸) or the recently released *AlphaFold-Multimer* (Evans *et al.*, 2021 ▸). In such cases tools such as *Coot* (Casañal *et al.*, 2020 ▸) or *ISOLDE* (Croll, 2018 ▸) make interactive model refinement and rebuilding relatively fast and simple, but subjective visual interpretation of a map by a user is still required. This process, although significantly simplified due to the availability of reliably predicted initial models, inevitably results in sporadic errors.

Errors may occur in every structure, regardless of the resolution or the best efforts of the experimentalist. However, they seem to be more common in cryo-EM, where models are often built quickly, under pressure and into reconstructions spanning a wide range of local resolutions. Although many issues can be corrected automatically (Joosten *et al.*, 2014 ▸; Liebschner *et al.*, 2021 ▸), visual residue-by-residue inspection by an experienced structural biologist remains the best way to judge the quality of a model. This, however, is time-consuming and requires a level of expertise that is rarely available (Croll, Williams *et al.*, 2021 ▸). Therefore, computational expert systems for model validation such as *MolProbity* (Chen *et al.*, 2010 ▸) are indispensable in the routine detection of modelling errors. Even these tools, however, usually require experience in separating severe problems that must be corrected from unusual features that may be left in a model. Moreover, finding an optimal way of correcting an issue is not always straightforward.

In cryo-EM, one of the most difficult problems to identify and correct are register-shift errors, where residues are systematically assigned the identity of a residue a few amino acids up or down in the sequence. When the resolution allows, register shifts can be identified using aromatic residues, which are usually well resolved in the density. This can conveniently be performed using a dedicated, interactive tool implemented in *ISOLDE*. The process, however, cannot easily be automated as map-fit measures are usually more sensitive to atoms outside density than to density left without a model (Croll, Williams *et al.*, 2021 ▸). Nevertheless, tools such as *EMRinger* (Barad *et al.*, 2015 ▸), *Q*-score (Pintilie *et al.*, 2020 ▸) and *SMOC* (Joseph *et al.*, 2016 ▸) can in principle be used to detect register shifts, even though there are no clear criteria that might be used to translate validation-score fluctuations to specific problems in a model. Moreover, these density-fit scores are strongly dependent on local resolution, which may hinder the recognition of register shifts from the effects of tracing problems or variations of local resolution.

Register-shift errors may also have an effect on backbone geometry when a number of side chains are forced into density volumes that are too small. It has been shown that these can in principle be detected using *CaBLAM* (Richardson *et al.*, 2018 ▸). Moreover, the very source of register shift, which is often a backbone-tracing issue (for example a deletion or insertion), can be occasionally detected based on backbone-geometry problems (Lawson *et al.*, 2021 ▸).

Neverthess, to the best of our knowledge there is no tool available that has been developed specifically for the automated detection of register-shift errors in macromolecular models. Although available model-geometry and density-fit validation tools can in some cases help to detect these, there are no clear rules of thumb that would allow the selection of troubled regions that need to be carefully checked by a user. Furthermore, none of these tools can automatically suggest a possible fix to a plausible register-shift problem.

Here, we present *checkMySequence*, a new tool for the automated detection of register-shift errors in cryo-EM models. The method is based on *findMySequence*, a protein sequence-identification tool for crystallography and cryo-EM (Chojnowski *et al.*, 2022 ▸). The *findMySequence* program uses a neural network classifier to predict residue-type probabilities for a backbone-only protein model and a corresponding map. Based on the predicted probabilities, the program can either identify the most plausible sequence for a given model in a sequence database or assign model fragments to a specific target sequence. The *checkMySequence* algorithm uses tools implemented in *findMySequence* to assign input model fragments to a reference sequence. It identifies regions where the new sequence assignment challenges the sequence-assignment hypothesis in the input model. This approach provides a conceptually simple, fast and intuitive tool for the reliable detection of register shifts in cryo-EM models, including very large macromolecular complexes (for example complete ribosomes). If an issue is detected, the method suggests a more plausible sequence assignment to the user. We show that *checkMySequence* can reliably identify register-shift errors in models deposited in the Protein Data Bank (PDB; Berman *et al.*, 2000 ▸) that have already been reported in the literature and a number of new, previously unidentified errors.

## 2. Model validation with a systematic sequence assignment

**Figure 1:**
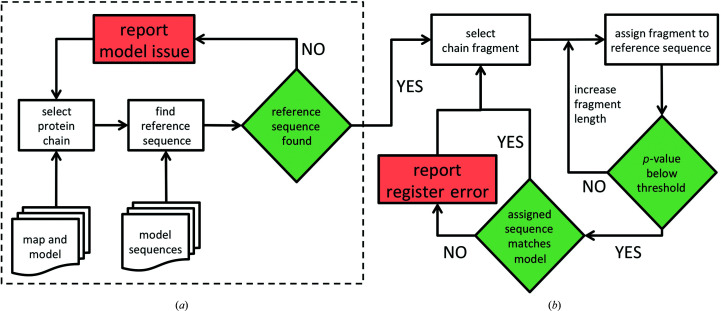
Flowchart of a model-sequence validation procedure. The dashed box in (*a*) encloses the initial steps of reference-sequence identification for each protein chain in the input model. If a reference sequence can be identified, the chain is divided into continuous test fragments that are systematically assigned to the reference sequence (*b*). The method reports possible issues (red boxes) if a reference sequence cannot be identified or if a sequence assigned to a test fragment with high confidence (*p*-value below the threshold) does not match the input model.

The method requires the input of a cryo-EM map, a corresponding atomic model and sequences of all of the model chains. Initially, for each protein chain in the input model the method identifies a reference sequence. It uses a protocol implemented in the *findMySequence* program (Chojnowski *et al.*, 2022 ▸) based on a neural network residue-type classifier and the *HMMER* (Eddy, 2011 ▸) sequence-comparison suite (Fig. 1 ▸ *a*). Each chain for which a reference sequence can be identified is divided into continuous overlapping test fragments that are systematically assigned to the reference sequence. The program identifies the most plausible assignment of a test fragment to a reference sequence given residue-type probabilities estimated from a map and backbone coordinates. We assume here that the test fragments are continuous and ignore all modified residues in the model. To account for variations in the accuracy of residue-type probability estimates and different lengths of reference sequences, for each assignment we estimate a *p*-value, or a probability that it was observed by chance. Additionally, to compensate for lower local resolutions, the initial fragment length of 20 residues is increased to a maximum of 60 residues if the corresponding sequence-assignment *p*-value exceeds a threshold defined in Section 4.1 (Fig. 1 ▸ *b*). Depending on the result of the initial reference-sequence identification and a test-fragment sequence-assignment procedure, the following alternative outcomes are possible.

(i) The reference sequence for a chain cannot be identified; the corresponding sequence is missing in the input, the chain is traced in a very low local resolution region or is mistraced.
(ii) The sequence assignment of a test fragment is unreliable (the *p*-value is above the threshold defined in Section 4.1); there is not sufficient evidence to confirm or reject the corresponding input model sequence.
(iii) The sequence assignment of a test fragment is confident (the *p*-value is below the threshold) and the assigned sequence agrees with the input model; the corresponding input model sequence assignment is confirmed.
(iv) The sequence assignment of a test fragment is confident (the *p*-value is below the threshold), but the assigned sequence does not agree with the input model; there is a plausible register error in the corresponding input model.

## 3. Materials and methods

### 3.1. Protein model benchmark set

Atomic model coordinates of macromolecular structures determined using cryo-EM were downloaded from the PDB as of 20 August 2021 together with the corresponding reconstructions and reference sequences. Only structures determined at a resolution of 4 Å or better, with a molecular weight below 500 kDa and with half-maps available for download in EMDB (Velankar *et al.*, 2016 ▸) were considered. A total of 796 structures fulfilled these criteria. For each of the half-map pairs local resolution maps were calculated using *RESMAP* version 1.1.4 (Kucukelbir *et al.*, 2014 ▸) with default parameters.

### 3.2. Protein-chain test-fragment selection

Continuous protein-chain test fragments were selected by shifting a ‘focus window’ of fixed length in steps of five residues along all protein chains in a model. For the benchmarks we used test fragments of 20 and 40 residues. Only chains with at least 95% standard amino-acid residue content were considered. If multiple conformations of a residue were present in a model, only the first one was processed. The mean local resolution of the selected chain fragments was calculated for the grid points of a corresponding local resolution map within 2 Å of any atom in a fragment. A random coordinate shift was applied for benchmarks only to all model atoms independently, ignoring any stereochemical restraints.

### 3.3. Map preprocessing

Before use, the input map resolution was truncated in reciprocal space to 2.5 Å to account for the absence of cryo-EM reconstructions determined at ultrahigh resolution in the residue-type classifier training set implemented in *findMy­Sequence*. Map blurring and sharpening were performed in reciprocal space and implemented in the *checkMySequence* code using tools from the *cctbx* library.

### 3.4. Implementation and availability

The sequence-validation program *checkMySequence* was developed based on routines implemented in *findMySequence*. It was developed in Python 3 with extensive use of the *PyTorch* (Paszke *et al.*, 2019 ▸), *NumPy* (Oliphant, 2006 ▸), *SciPy* (Virtanen *et al.*, 2020 ▸), *cctbx* (Grosse-Kunstleve *et al.*, 2002 ▸) and *CCP*4 (Winn *et al.*, 2011 ▸) libraries and utility programs. For making sequence database queries, we use the *HMMER* suite version 3.3.2. The program source code and installation instructions are freely available under a BSD-3 licence at https://gitlab.com/gchojnowski/checkmysequence.

## 4. Results and discussion

### 4.1. Sequence-assignment validation: finding a better hypothesis

Our working hypothesis is that the input model sequence is correct and agrees with an unknown, ground-truth reference model. If a result of the sequence-assignment procedure is conclusive, the hypothesis can be either confirmed or challenged by providing a model that explains the cryo-EM map features better. This approach, however, requires clear criteria for assessing the statistical significance of the sequence-assignment results.

The sequence-assignment procedure used in this work has been calibrated to provide a *p*-value estimate: a probability that the result was obtained by chance. In the current setup, however, we have no means of validating the reliability of this in detail as the reference structures available in the PDB may, and do occasionally, contain sequence-register errors that can only be identified and corrected by detailed inspection by an experienced modeller. Indeed, in a recent group effort, the members of the Coronavirus Structural Task Force were able to identify multiple tracing and sequence-assignment errors in a relatively small, representative set of PDB-deposited *Sarbecovirus* protein models (Croll, Diederichs *et al.*, 2021 ▸).

To address the issue of benchmark set reliability, we decided to undertake a large-scale, nonparametric approach assuming that errors in deposited PDB models are scarce and will weakly affect the overall conclusions. We tested the agreement with reference models of sequences assigned to continuous protein-chain fragments of 20 amino acids systematically selected from 796 protein models from the benchmark set described in Section 3.

**Figure 2:**
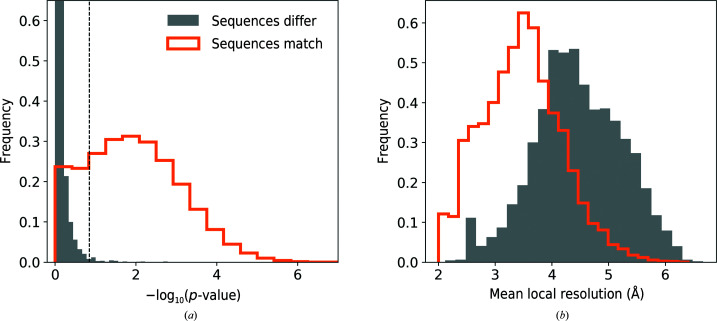
Comparison of sequence-assignment results for 166 713 protein-chain test fragments of 20 residues from the benchmark set. Distribution of (*a*) *p*-value and (*b*) mean local resolution of test fragments for which the assigned sequence matches and differs from the reference model. The dashed line in (*a*) corresponds to a 99.5% one-sided confidence interval estimated for fragments with an assigned sequence that differs from the reference.

For a total number of 166 713 protein-chain test fragments for which a reference sequence could be identified (see Section 2 for details), the assigned sequence matched the corresponding model in 156 091 (94%) and differed in 10 622 (6%) of cases. Protein-chain fragments with assigned sequence matching the reference and different from the reference are well separated by the corresponding *p*-value (Fig. 2 ▸ *a*). Indeed, a one-sided 99.5% confidence interval for fragments with a sequence assignment that does not match the reference (dashed line in Fig. 2 ▸ *a*) corresponds to 13% of cases with matching sequences. The relatively large number of fragments assigned a correct sequence with a high *p*-value may be due to the presence of model stretches built into local resolution regions that are too low for reliable sequence assignment. Indeed, the distribution of correctly assigned sequences is clearly shifted towards better local resolutions compared with assignments with incorrect sequence (Fig. 2 ▸ *b*). These observations clearly show that the *p*-value is a reliable criterion of the validity of the sequence-assignment procedure. Moreover, as expected, if sequence-assignment errors are present in the benchmark set models then they are relatively rare.

In this work, we treat as conclusive sequence assignments with a *p*-value outside the 99.5% one-sided confidence interval estimated for fragments with assigned sequence mismatch (Fig. 2 ▸ *a*). Although this choice of threshold is arbitrary, it corresponds to results that are very rare in our benchmark set and may indicate an outlier. We will show later that many sequence assignments outside this *p*-value interval are indeed due to plausible reference-structure errors.

### 4.2. Compensating for low local resolution

**Figure 3:**
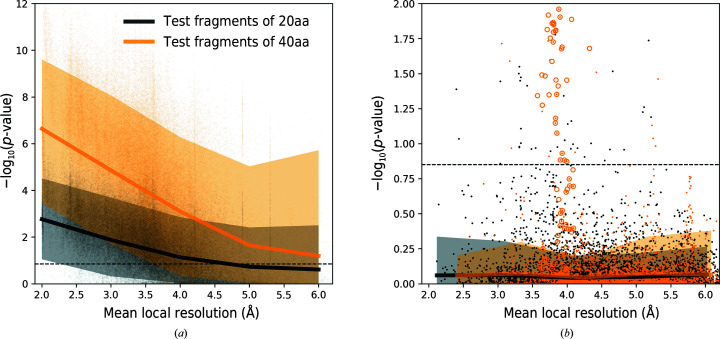
Medians and 90% confidence intervals for the sequence-assignment *p*-value as a function of local resolution for protein-chain test fragments of 20 and 40 amino-acid residues (20aa and 40aa, respectively). (*a*) shows fragments with sequences matching the reference. Fragments for which the assigned and reference model sequences differ are presented in (*b*). The dashed line corresponds to a 99.5% one-sided confidence interval estimated for fragments of 20 amino acids with an assigned sequence that differs from the input model sequence. Orange circles depict an outlier that is discussed in the text (the cytoplasmic domain of a transient receptor potential channel; PDB entry 6cv9). The ordinate axes show −log(*p*-value) for the test fragments; higher values correspond to lower *p*-values and more reliable sequence assignments.

In the previous section we noted that observed protein fragments with correctly assigned sequence and a high *p*-value may correspond to map regions with a local resolution that is too low for *de novo* tracing. To further investigate this issue, we plotted the sequence-assignment *p*-value as a function of the mean local resolution of the corresponding test fragments. The *p*-values are clearly higher for test fragments modelled into lower local resolution regions (Fig. 3 ▸ *a*), and half of the fragments exceed the threshold defined in the previous section at resolutions as low as 6 Å. At the same time, we observed that the *p*-values for sequence assignments that do not match the reference are independent of local resolution (Fig. 3 ▸ *b*). This clearly indicates that sequence misassignment is often related to intrinsic properties of a model (for example tracing errors) and not to low information content of a corresponding map region. We also observed that the *p*-value gap between correct and incorrect sequence assignments increases for longer fragments. This can be used to compensate for lower local resolution.

### 4.3. The effect of coordinate errors

**Figure 4:**
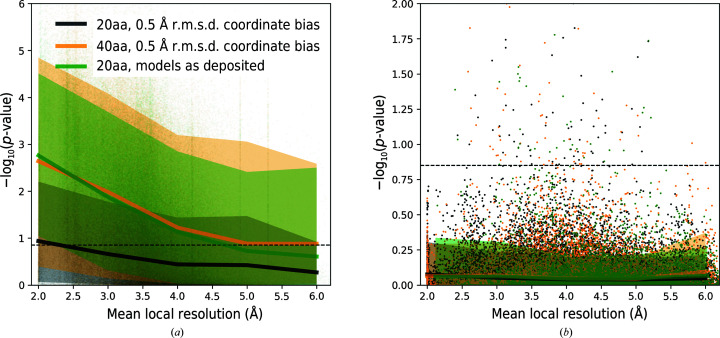
Medians and 90% confidence intervals for the sequence-assignment *p*-value as a function of local resolution. Data are shown for protein-chain fragments of 20 and 40 amino-acid residues (20aa and 40aa, respectively) for models as deposited and in the presence of artificial coordinate bias. (*a*) shows fragments with sequences matching the reference. Fragments for which the reassigned and reference sequences differ are presented in (*b*). The dashed line corresponds to a 99.5% one-sided confidence interval estimated for fragments of 20 amino acids with an assigned sequence that differs from the corresponding input model sequence. The ordinate axes show −log(*p*-value) for the test fragments; higher values correspond to lower *p*-values and more reliable sequence assignments.

To test the validity of our earlier observations in the presence of model errors, we plotted the sequence-assignment *p*-values for protein-chain test fragments at various levels of randomization of atom coordinates. We observed that coordinate randomization increases the *p*-values for all fragments, regardless of whether their assigned sequence matches or does not match the reference model (Fig. 4 ▸). As a result, fewer fragments exceed the *p*-value threshold defined in the previous section (Fig. 2 ▸ *a*), which reduces the predictive power of the presented approach. Nevertheless, we observed that the negative effect of coordinate errors can be compensated with longer test fragments (Fig. 4 ▸ *a*).

### 4.4. The effect of map sharpening and blurring

In earlier work, we observed that many deposited maps are oversharpened to a level that hinders their interpretation (Chojnowski *et al.*, 2021 ▸). As maps are often sharpened or blurred by microscopists based on subjective criteria, for example to increase the interpretability of specific map regions (Nicholls *et al.*, 2018 ▸), we decided to investigate the effect of excessive map processing on our sequence-assignment procedure.

**Figure 5:**
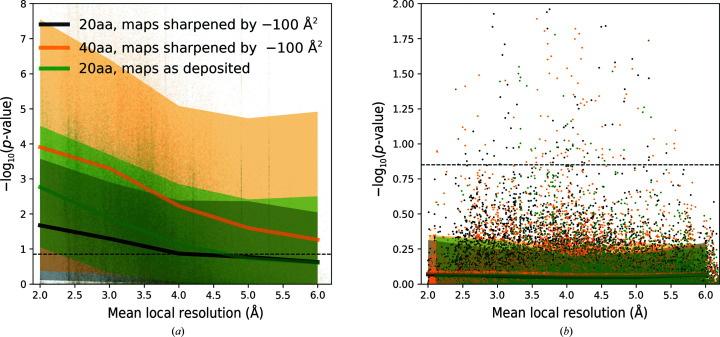
Median and 90% confidence intervals for the sequence-assignment *p*-value as a function of local resolution. Data are shown for protein-chain fragments of 20 and 40 amino acids (20aa and 40aa, respectively) and corresponding maps as deposited or sharpened by −100 Å^2^. (*a*) shows fragments with sequences matching the reference. Fragments for which assigned and reference-model sequences differ are presented in (*b*). The dashed line corresponds to a 99.5% one-sided confidence interval estimated for fragments of 20 amino acids with an assigned sequence that differs from the corresponding input model sequence. The ordinate axes show −log(*p*-value) for the test fragments; higher values correspond to lower *p*-values and more reliable sequence assignments.

We observed that excessive input map sharpening and blurring have a similar influence on sequence-assignment *p*-values (not shown). However, map sharpening (Fig. 5 ▸) has more impact on fragments built into high local resolution regions. We attribute this to the neural network residue-type classifier used in this work, which was trained on an unstratified set of deposited maps. It reflects the experimentally observed distribution of local resolutions, where very high local resolution regions may be underrepresented. We observed that the negative effect of excessive map sharpening or blurring (data for blurred maps were omitted for clarity) can easily be compensated with a longer test-fragment length (Fig. 5 ▸ *a*). We did not observe any effect related to map blurring or sharpening for test fragments with an assignment sequence that did not match a reference model.

### 4.5. Performance of reference-sequence identification

A crucial step in the described method is the identification of reference sequences for each chain in the input model. For this purpose, we use a procedure implemented in *findMy­Sequence.* The program uses a neural network classifier to produce residue-type probability profiles that are further used to query input sequence sets with tools from the *HMMER* suite. Sequence matches identified by *HMMER* are scored with an *E*-value: the number of expected hits in a comparable set containing only random sequences. The lower the *E*-value, the more reliable the corresponding match. Since this approach is inherently very sensitive to map and model quality, we investigated how coordinate errors and excessive map sharpening or blurring affects the performance of the reference-sequence identification procedure.

Overall, of 3378 protein chains longer than ten residues in our benchmark set, *checkMySequence* correctly identified 3162 (94%). For the remaining 216 structures the program returned no results, which is reported to a user as a plausible error. Indeed, we observed that many of them were clearly shifted outside corresponding cryo-EM reconstructions (for example PDB/EMDB entries 6vx7/EMD-21432, 6vp8/EMD-21250, 6tqm/EMD-10555 and 6xi8/EMD-22191).

**Figure 6:**
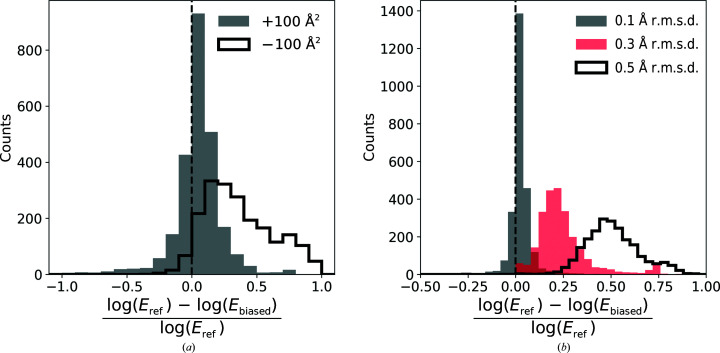
Relative change in *E*-value estimates for reference sequences identified by *checkMySequence* using the *HMMER* suite. The effect of (*a*) map blurring or sharpening (*B*-factor correction of +100 and −100 Å^2^, respectively) and (*b*) model coordinate bias. *E*_ref_ refers to *E*-values obtained for reference models and maps, whereas *E*_mod_ was obtained for the same map–model pairs after applying coordinate bias or map sharpening/blurring.

We observed that map processing increases *HMMER* *E*-values, but the effect of sharpening (negative *B*-factor correction) is more prominent than blurring (Fig. 6 ▸ *a*). There are also a number of structures for which map blurring significantly improves the performance of the method (lower *E*-values). Moreover, the overall number of 3162 chains in the benchmark set for which the reference sequence could have been identified reduces to 3149 for blurred maps and to 2508 for sharpened maps. This is in line with an earlier observation that many deposited maps are heavily oversharpened (Chojnowski *et al.*, 2021 ▸). Interestingly, we also observed that coordinate bias reduces the number of identified sequences less than excessive map sharpening (Fig. 6 ▸ *b*). The initial number of recognized chain sequences decreases to 3151, 3100 and 2797 for all-atom r.m.s.d.s of 0.1, 0.3 and 0.5 Å, respectively. Nevertheless, the identified sequences were always correct, regardless of map processing or model bias.

### 4.6. A register shift in an RNA-dependent RNA polymerase complex

The RNA-dependent RNA polymerase (nsp12) is essential for replication of the SARS-CoV-2 viral genome. In a recent study, a cryo-EM structure of nsp12 with two accessory proteins, nsp7 and nsp8, was determined at a resolution of 2.5 Å (PDB entry 7bv2/EMDB entry EMD-30210), providing valuable structural details on the mechanism of action of the antiviral drug remdesivir (Yin *et al.*, 2020 ▸). Due to its importance, the structure has been carefully analysed by the structural biology community.

**Figure 7:**
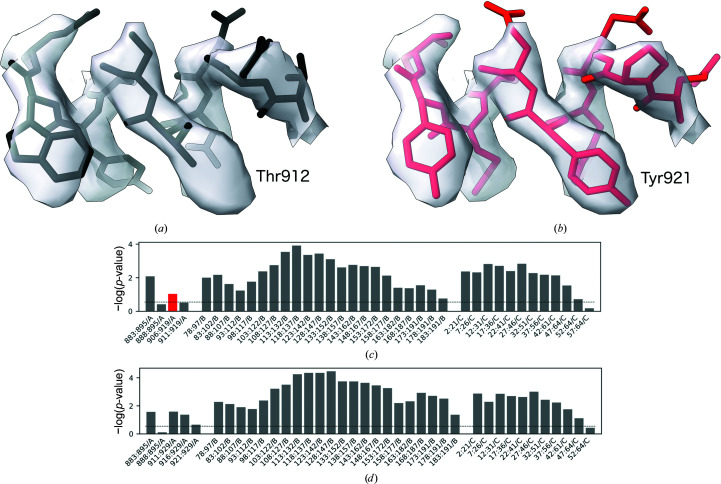
Fragment of an RNA-dependent RNA polymerase model. (*a*) Originally deposited version (PDB entry 7bv2/EMDB entry EMD-30210, residues A/908–A/917) and (*b*) the model deposited by the authors after applying a register-shift error that was also suggested by *checkMySequence* (residues A/917–A/926). The bottom panels depict standard *checkMySequence* graphical output for the (*c*) originally deposited and (*d*) corrected models. Grey bars represent fragments for which the assigned sequence matches the model or has a *p*-value higher than the threshold (dashed line). The red bar in (*c*) represents a fragment with register error, part of which is shown in (*a*). The bar plots show −log(*p*-value) for the test fragments; higher bars correspond to lower *p*-values and more reliable sequence assignments (be they correct or erroneous).

**Figure 8:**
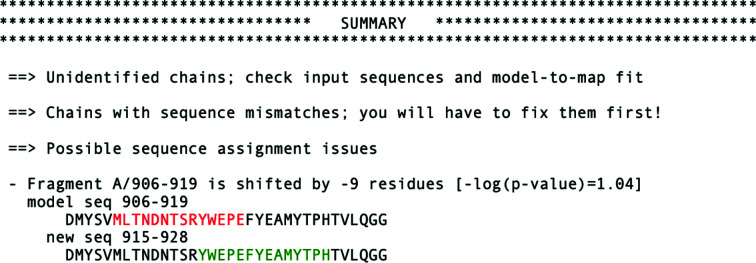
Standard text output of *checkMySequence* for the originally deposited version of the RNA-dependent RNA polymerase model (PDB entry 7bv2/EMDB entry EMD-30210). The program prints possible issues with unidentified reference sequences, sequence mismatches and register errors.

The original structure was shown to contain a number of potential problems that were quickly corrected in the updated PDB deposition (Croll, Williams *et al.*, 2021 ▸). One of the problems was a register shift of an isolated and relatively short (15 amino acids) α-helical fragment at the nsp12 C-terminus (Fig. 7 ▸ *a*). A close inspection of the fragment shows, for example, that Thr912 in the original model is too small for the corresponding side-chain density, which can be better explained with Tyr921 after shifting the model register by nine residues. This could not have been detected by a density-fit measure as the issue did not result in prominent density outliers. Similarly, as the whole fragment is register shifted there are no backbone geometry issues at the flanks of the shifted stretch. Nevertheless, the issue can be easily spotted with *checkMySequence* as a clear sequence-assignment outlier (red bar in Fig. 7 ▸ *c*). Moreover, a new sequence assignment suggested in the text output of the method could have been used to correct the issue, as it agrees with the updated coordinates (Fig. 8 ▸).

### 4.7. Cytoplasmic domain of a transient receptor potential channel

In the benchmark set, we identified a number of clear outliers where fragments were assigned sequences different from the reference model with low *p*-values. Particularly interesting was a cryo-EM structure of transient receptor potential cation channel subfamily C member 6 (TRPC6) determined at 3.8 Å resolution (PDB entry 6cv9/EMDB entry EMD-7637; Azumaya *et al.*, 2018 ▸). A number of fragments from this model form a clear, very low *p*-value cluster with assigned sequences that differ from the reference (orange circles in Fig. 3 ▸ *b*).

**Figure 9:**
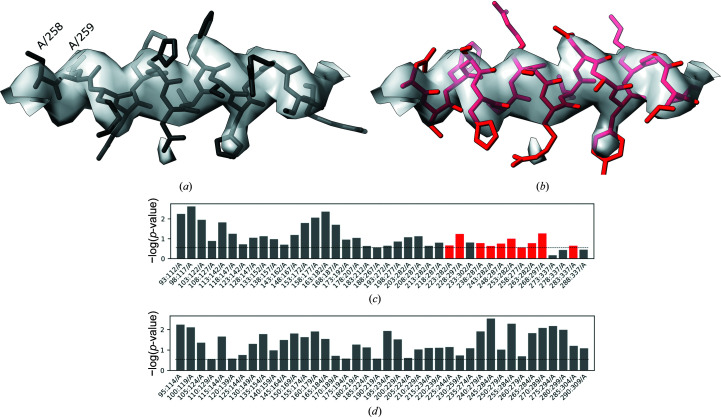
Fragment of the mTRPC6 cytoplasmic domain at 3.8 Å resolution (*a*) deposited in the PDB (PDB entry 6cv9/EMDB entry EMD-7637, residues A/258–A/272) and the same fragment rebuilt using *AlphaFold*2 prediction (Q61143, residues A/264–A/280; the pLDDT for this region exceeds 90). The bottom panels depict the corresponding *checkMySequence* graphical outputs for the (*c*) originally deposited and (*d*) corrected models. Grey bars represent fragments for which the assigned sequence matches the model or has a *p*-value higher than the threshold (dashed line). The red bars in (*c*) represent fragments with plausible register errors, including the α-helix shown in (*a*). The bar plots show −log(*p*-value) for the test fragments; higher bars correspond to lower *p*-values and more reliable sequence assignments.

Despite the relatively low resolution, due to the lack of any closely homologous structure the original model was built *de novo* into the map using *Coot*, which was a very challenging task. The overall fold of the structure was later confirmed by a number of closely related human TRPC6 structures determined at better resolutions. Nevertheless, we noticed a possible register shift in a linker helical domain of the structure (Fig. 9 ▸ *a*). As rebuilding the structure *de novo* would be very difficult owing to the relatively low local resolution, we decided to use a corresponding *AlphaFold*2-predicted structure database (Varadi *et al.*, 2022 ▸) to interpret the map (ID Q61143). The relevant region of the predicted model was fitted into the EM reconstruction using the ‘Jiggle-Fit this molecule with Fourier Filter’ tool and refined in real space with all-molecule self-restraints at a 5 Å distance cutoff using *Coot* version 0.9.2-pre (Casañal *et al.*, 2020 ▸), and was additionally refined using *phenix.real_space_refine* version 1.18.2 (Afonine *et al.*, 2018 ▸) with default parameters. Visual inspection of the new model showed a few clear signs of improvement in the match to the map in regions that resulted in a register shift in the original model [for example, the deletion in the left-hand end of the α-helix presented in Fig. 9 ▸(*a*), residues A/258 and A/259]. Nevertheless, we noticed no prominent signs of sequence-register improvement, for example for aromatic residues. The *checkMySequence* scores, however, improved significantly (Figs. 9 ▸ *c* and 9 ▸ *d*), which suggests that the new backbone model fits the corresponding map better and provides a better basis for a reliable sequence assignment. Moreover, the sequence register of the new model, which was shifted by eight residues relative to the deposited coordinates (Fig. 9 ▸ *b*), confirmed the *checkMySequence* suggestions. We also noted that the new model sequence register agrees with a closely related (94% sequence identity) human structure that has been determined recently at 2.8 Å resolution with much clearer side-chain densities in this region (PDB entry 6uz8; Bai *et al.*, 2020 ▸). These results show that *checkMySequence* can be used for the interpretation of maps and model validation at resolutions where visual map interpretation is very challenging.

## 5. Conclusions

Sporadic errors are an intrinsic part of the macromolecular model-building process. Modelling in cryo-EM reconstructions seems to be particularly affected as target structures are usually large, map resolutions are heterogeneous and the pressure to release models quickly is strong. Although a number of model-validation tools have been developed to date, many of them specifically for cryo-EM map interpretation, register shifts remain one of the most difficult problems to identify and correct.

Here, we have presented a new method, *checkMySequence*, for the fully automated identification of register-shift errors in protein models built into cryo-EM reconstructions. The approach relies on the systematic assignment of short protein model test fragments to the target sequence and works as a statistical test. If there is sufficient statistical evidence, the input model sequence can be challenged by providing a sequence that explains the features of a map better. Although the strength of the approach in principle depends on factors such as local map resolution, model quality and a level of map sharpening or blurring, we have shown that these factors can be successfully compensated by automated adjustment of the test-fragment length. This avoids the problem of bias related to user-provided parameters such as map resolution. The method also removes the need for the user to understand and interpret validation scores: the results clearly state whether or not the input model sequence assignment can be improved.

A limitation of this approach is its use of relatively long test fragments, which are required to compensate for the low information content of cryo-EM maps. At lower local map resolutions, the test-fragment length can be increased to 60 amino acids from the default of 20. This means that the source of a sequence-register problem needs to be identified by a user within this range using interactive methods. It must be stressed, however, that if a plausible register shift is detected the method also suggests a new sequence assignment, which simplifies the error correction. The model errors can be conveniently rebuilt using tools available in the related method *findMySequence*. The requirement for long test fragments, however, may also result in the method missing short, local register-shift errors, where a tracing issue is promptly compensated (for example a missing residue compensated by an additional residue). Although the minimum length of a detectable register-shift stretch depends on the resolution, it should be generally longer than ten amino acids. Nevertheless, we show that the approach provides useful results at resolutions where a visual, residue-by-residue validation would be very challenging.

Apart from register-shift errors, the program also checks the input model for sequence mismatches (single-residue differences between the model and target sequence) and problems with residue numbering (for example continuous residue numbering ignoring gaps in a model) (Fig. 8 ▸). Another advantage of the presented method is its performance, which makes it readily applicable to the analysis of very large models. For example, validation of a complete 70S ribosome structure at 3.0 Å resolution (PDB entry 5we4/EMDB entry EMD-8814), with 58 protein chains and over 6400 protein residues, takes less than 3 min on a basic laptop. As we have shown previously (Chojnowski *et al.*, 2022 ▸), this structure contains several register-shift errors of the kind which could be avoided in other cases in the future by the use of *checkMySequence*. This is particularly important as detailed and exhaustive residue-by-residue analysis of such large models is rarely possible. In this context, the transparent graphical visualization (lower panels in Figs. 7 ▸ and 9 ▸) and clear text summary of the validation results in the command-line output of *checkMySequence* (Fig. 8 ▸) should be of particular interest to users.
